# E-cadherin roles in animal biology: A perspective on thyroid hormone-influence

**DOI:** 10.1186/s12964-016-0150-1

**Published:** 2016-11-04

**Authors:** María Fernanda Izaguirre, Victor Hugo Casco

**Affiliations:** Laboratorio de Microscopia Aplicada a Estudios Moleculares y Celulares, Facultad de Ingeniería (Bioingeniería-Bioinformática), Universidad Nacional de Entre Ríos, Ruta 11, Km 10, Oro Verde, Entre Ríos, Argentina

**Keywords:** E-cadherin, Adherens junction, Epithelia, Development, Evolution, Thyroid hormones

## Abstract

The establishment, remodeling and maintenance of tissular architecture during animal development, and even across juvenile to adult life, are deeply regulated by a delicate interplay of extracellular signals, cell membrane receptors and intracellular signal messengers. It is well known that cell adhesion molecules (cell-cell and cell-extracellular matrix) play a critical role in these processes. Particularly, adherens junctions (AJs) mediated by E-cadherin and catenins determine cell-cell contact survival and epithelia function. Consequently, this review seeks to encompass the complex and prolific knowledge about E-cadherin roles during physiological and pathological states, particularly focusing on the influence exerted by the thyroid hormone (TH).

## Background

Since the early 70s, prolific and significant research and hypotheses subscribe to the theory that unicellular to multicellular transition has occurred more than once during evolution through different genetic mechanisms for each kingdom in the tree of life. Particularly, cadherin and integrin cell adhesion molecules played a crucial role in metazoan transition [78, 171].

In addition, the interpretation of the cellular role of cadherin has changed over time, from a static membrane protein involved in cell-cell adhesion mechanic events to a membrane receptor involved in very dynamic intracellular signal transductions. These processes would be mediated not only from cell-cell junction platforms, but also from single receptors [29, 189, 216].

In different physiological contexts during development and adulthood, this adhesion is highly plastic, suffering remodeling due to numerous and complex signaling cascades finely coordinated in time and space. Thus, in pathological states such as cancer, the configuration of this adhesion is altered by genetic and epigenetic changes, resulting in modifications in the signaling pathways, loss of inhibition by contact, cell migration, and altered stromal interactions. A key group of these cell-cell adhesion molecules is the superfamily of cadherins, whose prototypical member is E-cadherin. This molecule was found for the first time in epithelial tissues [177, 215] and has been characterized as a potent suppressor of invasion and metastasis in studies dating back to 1990 (reviewed by [199]). E-cadherin plays a key role in determining cell polarity and differentiation, and thereby in the establisment and maintainance of tissue homeostasis. It is also important during development and during the whole life cycle of pluricelular organisms, mainly metazoans.

In this work, four major aspects bring E-cadherin into focus. First, epithelia are composed of cell phenotypes which exhibit the maximum polarity and whose epithelial identity is primarily specified by E-cadherin. Second, E-cadherin is involved in numerous processes such as, cell phenotype selection and migration, and morphogenetic movements occurring during metazoan development [26, 192, 193, 196]. Third, epithelia are usually located in direct contact with mutagenic and/or carcinogenic agents, so in men, 85–90 % of the cases can be attributed to epithelial cancers. Lastly, the complexity of in vivo animal studies has partly concealed the full spectrum of E-cadherin functions. Owing to the latter, the work-goal focuses on E-cadherin control mediated by thyroid hormones in a scarcely studied physio-pathological scenario.

## Review

### E-cadherin function during metazoan development

Cadherins are a huge family of Ca^2+^-dependent cell surface adhesion glycoproteins, which are differentially expressed in tissues and ontogeny among broad groups of organisms, from unicellular choanoflagellates to invertebrates and all vertebrates classes. Therefore, cadherin evolution has special relevance for understanding metazoan origins [2, 62, 66, 77, 78, 80, 140, 143, 171, 192, 196].

Classical cadherins, like E(epithelial)-cadherin, bind to β-catenin via the cytoplasmic domain (CD) and promote cell-cell adhesion [174] or are rapidly degraded [32, 76, 86]. As development progresses, cadherins establish and maintain AJs through adaptor proteins to the cytoskeleton [63, 64, 129, 141]. These complex structural units are called cadhesomes [216], and act integrating signals from extracellular and intracellular environments [19, 62, 85–87, 138]. In vertebrates, E-cadherin mRNA and protein are maternally expressed, beginning their zygotic expression from 2-cell embryo or gastrulation [8, 9, 34, 91, 114, 191]. Although E-cadherin is principally expressed in the epidermal ectoderm [191], it has also been found in derivatives of neuroectoderm, mesoderm and endoderm [39, 85, 87]. In some species, embrionary E-cadherin is necessary for 8-cell stage compaction, for trophectoderm expansion [7, 108], and for maintaining epiboly integrity [115]. Later during development, E-cadherin contributes to the morphogenesis of endodermal and neuroectodermal derivates [83], and digestive tract, kidney and skin remodeling [85–87, 179, 197]. In invertebrates, a vertebrate E-cadherin homolog is critical for cell rearrangement [198] and packing geometry [35].

Some studies suggest that three modern cadherin families —lefftyrins, coherins, and hedglings— were present in the last common ancestor of choanoflagellates and metazoans, and they may have evolved to diverse metazoan signaling and adhesion gene families [140]. Several cadherin-analogous functions have been hypothesized in non metazoan unicellular lineages in spite of the fact that cadherins are undocumented in choanoflagellates. These organisms express members of key cell signaling and adhesion protein families that were previously thought to be exclusively found in animals [97, 98]. By studying cell differentiation and development in some choanoflagellates, it may be possible to characterize some ancestral functions (bacterial prey adhesive capture, attachment to environmental substrates, gamete recognition and hormonal signaling) of proteins that regulate animal development [46, 47, 98]. Interesting, numerous studies show interaction processes between bacteria or yeast and metazoan cadherins promoting host invasion [17, 36]. In addition, other studies inform that no receptors or ligands were identified from the nuclear hormone receptor, WNT and TGF-β signaling pathways [98]. As we will explain later, the absence of nuclear hormone receptors and ligands, and molecules involved in Wnt signaling pathways suggest combined features and roles in a single molecule-type: the metazoan cadherins.

Taken together, these data and our results on TH-dependent E-cadherin control [56, 85], support the idea that classical cadherins could have emerged in the premetazoan to metazoan transition to respond to signaling mediated by nuclear hormone receptors and cytoskeleton connexions. Indeed, the development of complex multicellular organisms requires a genetic program regulated by nuclear hormone receptor signaling and dynamic cytoskeleton reorganizations.

### E-cadherin structure, function and dysfunction

**Fig. 1:**
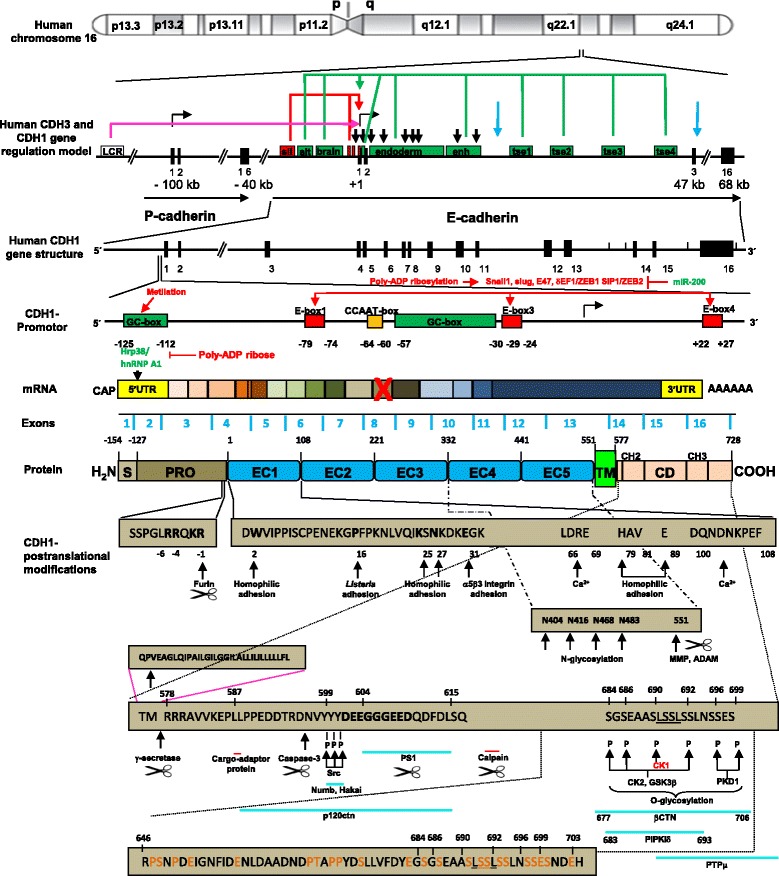
Structure and regulation of E-cadherin gene and protein. Scheme represents human chromosome-16q22.1 cadherin cluster (CDH1/E-cadherin and CDH3/P-cadherin locus), and a regulation model both CDH1 locus and protein. CDH1 locus has 16 exons (*black bars*), *cis*-regulatory elements (DHSs, *vertical arrows*), transcription and translation start sites (*small horizontal arrows*), several enhancer sequences (*green boxes* and *arrows*) ─alternative intron 2-independent gene activation in late embryogenesis (alt), specific expression in (brain) or endoderm (enh), or ectoderm/tissues (tse1-4)─, downregulated sequences (*red arrows*) ─E-boxes and brain-specific silencer (sil)─, and yolk sac-specific elements outside of intron 2. Locus control region (LCR) could activate or downregulate gene activity (*purple arrow*). CpG methylation and E-box-bounded specific repressors (Snail1, slug, E47, δEF1/ZEB1 SIP1/ZEB2) control the promoter, as well as poly-ADP ribosylation and repressor inhibition (miR-200). MIR and MaLR regulatory-associated repetitive elements (light blue arrow) were bioinformatically found in introns 2 and 3, and are involved in exonization and increased novo intronic transcription. New transcripts have been revealed from intron 2-transcription and exon 11 skipping (*red cross*). In *Drosophila melanogaster*, CDH1-mRNA translation is suppressed by poly-ribosylation of HnRNP attached to E-cadherin 5’UTR. Human CDH1 pro-protein harbors topological domains: signal peptide (S), pro-peptide (PRO), extracellular with cadherin repeats EC1-EC5 domains, transmembrane (TM, with proximal CH2 and distal CH3), cytoplasmic domain (CD); binding sites of delta1-catenin and presenilin-1 (PS1), p120-ctn, β-catenin (βCTN), calcium; ubiquitination and short intracellular half-life sites rich in proline (P), glutamic acid (E), serine (S) and threonine (T) (PEST); motif highly conserved (Leu-Ser-Ser-Leu); acidic residues cluster of endocytic signal (DEE 602–604). Cleavage sites by proteases (*scissors*): metalloprotease (MMP), gamma-secretase/PS1 (presenilin-1) and caspase 3. Inhibitory or stimulatory phosphorylation sites for casein kinase-1 (CK1) (*red*) and CK2, GSK3β and PDK1 respectively. N-glycosylation at Asn-483 is essential for expression, folding and trafficking. *Ligth blue horizontal lines* indicate protein binding domains to E-cadherin.

In their mature state, most cadherins have three segments: the extracellular (EC), trans-membrane(TM) and cytoplasmic (CD) domains (Fig. 1). The EC domain is mainly involved in homophilic recognition and Ca^2+^-dependent adhesion mechanisms, and it is constituted by a variable amount of cadherin type-repeats (EC_n_). Each one has a β-sandwich Ig-like folding that contains conserved Ca^2+^-coordinating regions [15]. Each EC_n_ is enumerated from the outermost N-terminal EC1 to the closest to membrane EC1 + n. The CD domain exhibits the highest variability among the cadherin subfamilies [66, 77] and binds to different intracellular proteins giving both functional connection to the cytoskeleton [15] and diversity [66, 140].

#### E-cadherin function control via EC

The mature classical E-cadherin is a single-pass transmembrane glycoprotein with five ectodomains (EC1–5), each made up of ~110 amino acid residues. Their NH2-terminal ectodomain mediates adhesive binding to cadherins present on the surfaces of neighboring cells. Their four interdomain junctions are characterized by three calcium binding sites ─DXD, DRE, and DXNDNAPXF sequence motifs─ [207]. Cadherin function requires calcium to rigidify the EC domain [27, 112, 146] and to stabilize the protein [156, 159] avoiding proteolysis [79, 190], and allowing its proper localization [57, 156, 159].

E-cadherin mediates principally in cell-cell homophilic interactions through self-recognition of the conserved histidine-alanine-valine (HAV) sequence within EC1 [14] (Fig. 1), and a strand-swapping interface. In this surface, a Tryptophan in position 2 (W2/Trp2) inserts into a hydrophobic pocket from another *cis* (from the same cell surface) or *trans* (from an adjacent cell) cadherin [100, 111, 182]. Although another *cis* dimerization has been postulated [15], several models disagree on the roles and numbers of the inner EC domains involved in cadherin homophilic interaction [31, 66, 112, 160].

Cleavage of the ~130 amino acids prodomain of immature cadherin represents the switch from a non adhesive to the functional form [67]. Even though the specific E-cadherin endoprotease has not been identified yet [145], the E-cadherin proprotein contains a furin-cleavable motif [SSPGLRRQKR] (Fig. 1) [73, 136], and sequence specificity for other mammalian convertases [161]. In contrast, mature E-cadherins are inactivated by cleavage in the EC domain mediated by matrix metalloproteinases [37] and others proteases [89, 153], in a process known as ectodomain shedding, thus promoting the invasion [44, 122, 187] (Fig. 1).

In addition, it has also been reported that E-cadherins can adhere heterophilically to integrins αEβ7 and α2β1 [30, 176, 205], killer-cell lectin-like receptors G1 [173], and numerous infectious agent proteins that target E-cadherins as an entry receptor [5, 36, 132].

#### E-cadherin function control via CD

In active functionally adhesion complexes E-cadherin associates with intracellular components forming AJs. E-cadherin CD interacts with several cytoplasmic proteins, being the catenins the best understood: β-catenin or γ-catenin, α-catenin, and p120-catenin (p120-ctn) [41, 128, 133, 134, 165]. A homolog of β-catenin, γ-catenin/plakoglobin [152], can substitute it under some circumstances [38]. When they are fully incorporated in complexes with cadherins, these three catenins associate with a stoichiometry of one of each catenin per cadherin molecule [90, 147]. β-catenin binds directly to the distal ~72 amino acids of the E-cadherin CD through a 30-amino acid “core”, anchoring indirectly to α-catenin (Fig. 1) [76]. P120-ctn binds independently to the ~29 amino acid membrane-proximal region of the cadherin CD [165, 199] (Fig. 1). α-Catenin associates with the actin cytoskeleton [167, 214] and numerous adaptor proteins to strengthen cell-cell adhesion [1, 199].

Several cleavage fragments of E-cadherin CD and disassembling of their adhesive complexes have been reported. During apoptosis or calcium influx, presenilin 1 (PS1)/γ-secretase cleaves between human E-cadherin residues Leu731 and Arg732 [127] and caspase-3 cleaves on site 747-DTRD-750 and releases a 25-kDa fragment [93, 183]. In addition, during tumoral progression calpain mediates E-cadherin proteolysis [168] (Fig. 1).

**Fig. 2:**
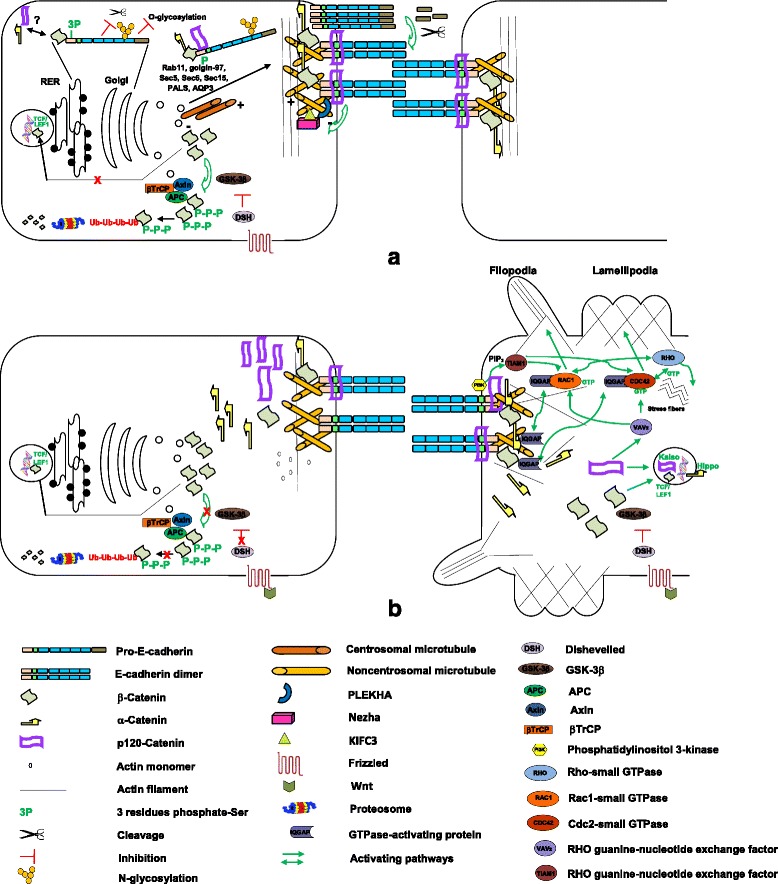
Signaling pathways involved in cell-cell adhesion mediated by E-cadherin in physiological and pathological conditions. **a** Shortly after their synthesis, cadherins associate with β-catenin and phosphorylation on the RER avoid degradation of uncomplexed cadherins and the pro-region cleavage by furin proteases in the trans-Golgi network. When the *cis* E-cadherin surface pool increases, pro-domain cleavage induces dimerization, *trans* homophilic bonding and E-cadherin adhesive activity. Delivery of newly synthesized E-cadherin to the basolateral cell surface should be mediated by Rab 11, golgin-97, Sec5, 6, Protein Associated with Lin Seven 1 (PALS), aquaporin 3 (AQP3), p120-ctn, and possibly β-catenin, via localization at the centrosome. **b** AJs-disassembly by dysfunction of E-cadherin─catenin complexes (CCC) releases catenins that accumulate in the cytoplasm. β-catenins are then sequestered and phosphorylated by the adenomatous polyposis coli (APC)–axin–glycogen synthase kinase 3β (GSK-3β) complex, inducing their ubiquitination by the E3 ubiquitin-ligase βTrCP subunit for proteosomal degradation. However, if at the same time the Wnt signaling pathway is activated, GSK-3β is repressed and β-catenins are no longer phosphorylated and are translocated to the nucleus where their bind TCF/LEF1 transcription factors and modulate gene expression involved in cell proliferation and migration. Cytoplasmic p120-ctns detached from AJs, in turn, activate Rac1 and Cdc42 through Vav2 (Rho-GEF) and represse Rho, promoting filopodia and lamellipodia projections. PI3K is recruited to the membrane by intact E-cadherin adhesion junctions, where it generates PIP3. This activates Tiam1 (Rho-GEF) and subsequently Rac1 and Cdc42, sequestring the GTPase-activating protein (IQGAP1), avoiding IQGAP-binding to β-catenins, and displacing α-catenins from the CCCs, thereby disrupting the CCC-anchoring to the cytoskeleton. Thus, while the activation of Cdc42 and Rac1 induces the formation of filopodia and lamellipodia respectively, Rho induces the formation of actin stress fibers. Cytoplasmic p120-ctn also can translocate to the nucleus to associate with Kaiso and modulate gene expression

It has been proposed that while β-catenin and plakoglobin facilitate indirect interactions between classic cadherins and the actin cytoskeleton at AJs in vivo, p120-ctn subfamily members induce the lateral (*cis*) clustering of cadherins [209], and the tethering of signaling or regulatory entities, such as kinases and phosphatases [118]. P120-ctn also stabilizes cadherins at the cell membrane by modulating cadherin membrane trafficking (endocytosis-recycling) and degradation [21, 139, 211]. Catenins are modified by kinases and/or phosphatases that are enriched at cell-cell contacts [6, 42, 52, 118, 203]. E-cadherins, in turn, are normally maintained in a tyrosine-dephosphorylated state through the action of phosphatases that are crucial for stabilizing AJs [51, 135]. In some cell contexts, phosphatase inhibition promotes release of E-cadherins and β-catenins from cell-cell contacts, enabling cytoplasmic catenins to relocate and function in the nucleus and promoting E-cadherin proteolysis [86]. Like phosphatase inhibition, receptor tyrosine kinase stimulation disassembles cell contacts mediated by cadherin─β-catenin─α-catenin [18, 203]. In addition, p120-ctn is an important modulator of RhoGTPase activities, such as RhoA, Rac1 and Cdc42 [3, 4, 142] and gene transcription [43, 95, 149, 162, 180]. On the other hand, p120-ctn interacts with tubulin influencing microtubule stability and dynamics, and thereby affects cell motility and directional migration independently of the cadherin adhesion system [81, 170] (Fig. 2).

As for α-catenin, it modulates the actin-cytoskeleton organization because it can occur either in a monomeric or a homodimeric form [13, 50, 65]. Upon the application of force on epithelia, the conformational change of monomeric αE-catenin uncovers the vinculin binding site, allowing vinculin to bind, and recruit additional F-actin to the cadherin-catenin complexes [126, 214]. Additionally, ZO-1, spectrin, vinculin [199] and Eplin [1] assemble F-actin to E-cadherin-catenin complexes. In parallel, the non-junctional cytosolic homodimeric αE-catenin pool inhibits the Arp2/3 complex, reducing membrane dynamics by preventing F-actin branch-formation [13].

### Dynamics of E-cadherin-mediated cell-cell adhesion

#### Establishment of adherens junctions

**Fig. 3:**
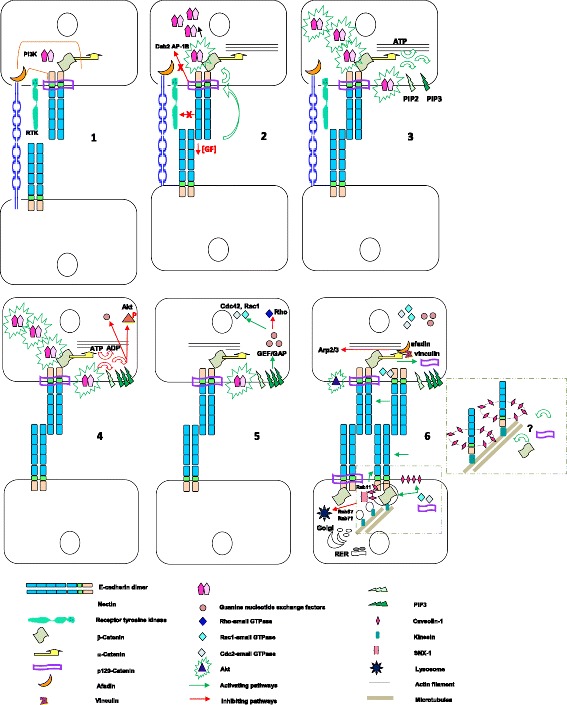
Dynamics of E-cadherin-mediated cell-cell adhesion Epithelial AJs are constructed on a foundation of homophilic contacts between E-cadherin clusters. Previous contacts between nectins inhibit non-trans-interacting E-cadherin endocytosis through afadin, Rap1, and p120-ctn, and increase their concentration at cell-cell adhesion sites. Immediately after E-cadherin trans-interaction, the junction complexes trigger activation of the phosphatidylinositol-3-kinase (PI3K)–Akt–protein kinase B pathway. Phosphatidylinositol-(3,4,5)-triphosphate (PIP3) is generated, and guanine nucleotide exchange factors are recruited to the membrane, activating Rac1 or Cdc42 and reducing Rho activation, which stimulates membrane and actin dynamics adjacent to the initial site of contact, increasing the probability of additional E-cadherin engagements. Alpha-catenin homodimerizes and is released from the cadherin-catenin complexes to bind at and antagonize with Arp2/3, facilitating the belt formation of unbranched actin filaments. While vinculin, afadin and alpha-actinin link with actin cytoskeleton, β-catenin and p120-ctn also link with tubulin cytoskeleton to route both vesicles of newly synthesized E-cadherin-catenin and E-cadherin-recycling-endosomes to the cell-cell contact sites. Down-stream of Rac and Cdc42, IQGAP1 binds β-catenin, which could localize in membrane ruffles and control cadherin internalization via SNX-1 preventing E-cadherin lysosomal degradation and recycling of E-cadherin back to the cell surface for AJ maintenance. P120-ctn binding covers cadherin juxtamembrane domain inhibiting RhoA locally and adaptor complex-binding that recruits cadherins into a coated pit. Thus, E-cadherin becomes withheld in plasma membrane junction domains. Meanwhile, PtdIns(3,4,5)P3 accumulation in the membrane signals for the formation and expansion of the baso-lateral surface, while Rac1 promotes cell polarity and lumen formation, cell cycle arrest of confluent epithelial cells, and survival of polarized epithelial cells. In parallel, E-cadherin downregulates ligand-dependent receptor tyrosine kinase activation, such as EGFR stabilizing cell-cell contacts. Insert: traffic of E-cadherin vesicles via p120-ctn or β-catenin coupled to kinesin for delivering to newly forming or remodeling junctions

Epithelial AJs are built on a foundation of homophilic contacts between (E or P)-cadherin clusters on the surface of adjacent epithelial cells [62, 117]. Cadherins alone are not sufficient for AJ formation; rather, cooperation between nectins and cadherins is required [166, 188] (Fig. 3). Appearently, the *trans*-interacting nectin inhibits non-*trans*-interacting E-cadherin endocytosis through afadin, Rap1, and p120-ctn, thereby further non-*trans*-interacting E-cadherin accumulates in the nectin-based cell-cell adhesion sites for AJ formation [74]. Once AJs have been established through intracellular partner binding to the cytoskeleton, E-cadherin contacts modulate actin filament organization at the underlying cortex [10, 50, 155] and microtubules network [130, 131].

It is known that catenins, including β-catenins, α-catenins, and p120-catenins, bind to immature E-cadherins while traveling through the endoplasmic reticulum and Golgi apparatus [40, 71]. Following synthesis in the rough endoplasmic reticulum and phosphorylation of the cytoplasmic domain, the E-cadherin pro-region is cleaved by furin proteases in the trans-Golgi network, an event that is mandatory for the mature cadherin to function in adhesion [59, 75, 116] (Fig. 2). Via golgin-97 dependent tubulovesicular carriers, E-cadherins leave the Golgi complex [124], and subsequently fuse with an intermediate recycling endosome, in route to the basolateral plasma membrane. This pathway for E-cadherins trafficking is tightly integrated with other proteins involved in epithelial polarity, such as Sec5, 6, and 15 [107, 193], Rab 11 [123], PALS (Protein Associated with Lin Seven 1), and aquaporin 3 (AQP3) [137], and it determines lumen formation [49] (Fig. 2).

The function and regulation of E-cadherins post-translational modifications in vivo have remained poorly defined. While the phosphorylations to eight serine residue cluster within a region that binds β-catenin modulate the β-catenin affinity and strengthen cell–cell adhesion [181], the E-cadherin cytoplasmic O-glycosylation (O-GlcNAc) blocks its cell surface transport, reducing intercellular adhesion [218]. In addition, E-cadherins have four consensus sites Asn-X-Ser/Thr for N-glycosylation in EC4 and EC5 domains [102], most of them conserved among species (Fig. 1). It has been established that, while the N-glycans at Asn 633/483 are essential for E-cadherin folding, trafficking, and proper expression [217], their modification with complex N-glycans weakens AJs [121, 157] (Fig. 2).

#### Signaling and maintainance of adherens cell-cell junctions

Chemical [3, 85, 86, 149] and mechanical [110, 214] signals from cell-cell junctions can be transduced through E-cadherin-CD via β-, α- and p120-catenin to the cytoplasm or nucleus. Mechanical stimulation generated by tumor cell proliferation leads to β-catenin phosphorylation via Src kinase at the site of its interaction with E-cadherin, increasing β-catenin nuclear localization, and upregulating the oncogenes Myc and Twist1 [204]. In physiological state, the formation of junctional complexes triggers activation of the phosphatidylinositol-3-kinase (PI3K)–Akt–protein kinase B pathway [151] because the PI3K-p85 subunit associates with AJs through direct binding to β-catenin [206] (Fig. 3). After recruitment of PI3K, phosphatidylinositol-(3,4,5)-triphosphate (PIP3) is generated, and guanine nucleotide exchange factors, that contain PIP3-binding pleckstrin homology domains, are recruited to the membrane and activate Rac1 [105, 210] or Cdc42 [94]. This stimulates membrane actin dynamics adjacent to the initial contact site, increasing the probability of additional E-cadherin engagements. α-Catenins, in turn, homodimerizes and are released from the cadherin─catenin complexes to bind at actin and antagonize Arp2/3 function, inhibiting actin branching and facilitating belt formation of unbranched actin filaments. Simultaneously, other actin-binding proteins such as vinculin, afadin and α-actinin link with actin cytoskeleton, and β-catenin and p120-ctn link with tubulin cytoskeleton to route vesicles of E-cadherin─catenin to the cell-cell contact sites. Meanwhile, PtdIns(3,4,5)P3 accumulation in the membrane signals for the formation and expansion of the basolateral surface, whereas Rac1 promotes polarity orientation and lumen formation. In tissue and cell-type specific contexts, Rac1 also triggers the activation of downstream signaling effectors, promoting cell cycle arrest of confluent epithelial cells, and survival of polarized epithelial cells [169]. Therefore, cadherins function in tissue morphogenesis by controlling both cell-cell adhesion and cell signaling. In this way, cadherins are involved in determining cell shape, position, migration [33, 68, 92, 125, 186], polarity [16, 200] and proliferation [96, 172], as well as tissular folding [201, 202]. In adherens junctions, E-cadherins and catenins determine cell-cell contact survival and epithelia function [11].

AJs are very dynamic structures that undergo constant remodeling. This can be low-scale remodelling involving replacement of individual or groups of molecules within the adhesive clusters without disrupting steady-state intercellular adhesions. It can also be large-scale junctional rearrangements that accompany breakdown and reformation of cell-cell contacts [82].

#### Growth factors (GFs) as specific regulators of E-cadherin─catenin traffic

**Fig. 4:**
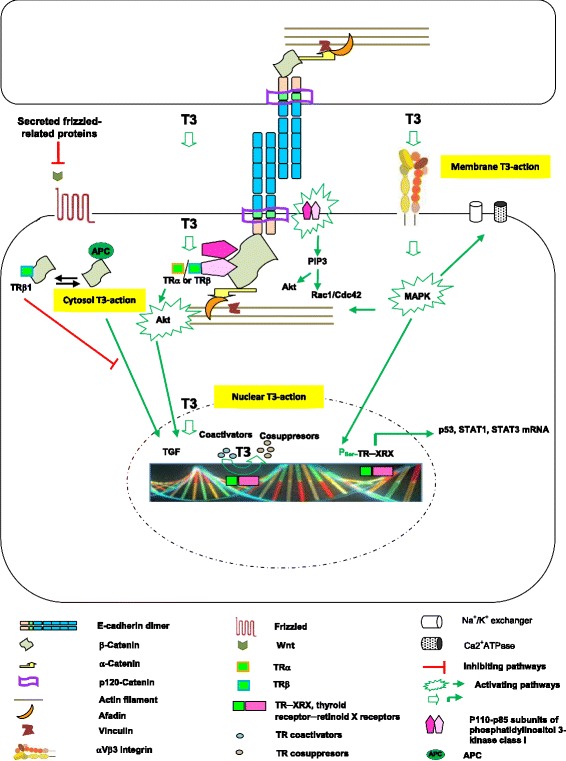
T3-actions from genomic and non-genomic effects on cell adhesion and differentiation during vertebrate development. THs modulate energy metabolism, growth and development by independent mechanisms. While thyroid calorigenesis is influenced predominantly via nuclear receptors, many of the TH effects over development are thought to be mediated via cytosolic and membrane partners. E-cadherin trans-interaction triggers activation of the phosphatidylinositol-3-kinase (PI3K)–Akt–protein kinase B pathway bound to β-catenin, generating phosphatidylinositol-(3,4,5)-triphosphate (PIP3), recruitment of guanine nucleotide exchange factors, activation of Rac1 or Cdc42 and Akt, and reduction of Rho activation. In addition, TRα or TRβ forms a cytoplasmic complex with the p85 subunit of PI3K, inducing protein kinaseB/Akt nuclear translocation and inhibition of the Wnt/β-catenin pathway through its interaction and consequent sequestration of β-catenin. The process results in down-regulation of cell proliferation. Simultaneously, TH binding to TRs causes heterodimerization with retinoid X receptors (RXRs), binding to T3 response elements located within the genomic regions and shooting target gene transcription. In the absence of T3, TRs interact with co-repressor proteins to inhibit target gene transcription. Following T3 binding, co-repressors are displaced and co-activator proteins are recruited to the ligand-bound TR complex, facilitating T3-dependent activation of the target genes. Besides the TR-mediated functions, THs also exert rapid non-genomic actions that are initiated at the cell membrane. Integrin αvβ3 is a specific membrane receptor for THs, which mediate activation of the mitogen-activated protein kinase (MAPK) intracellular cascade. TH-dependent MAPK activation subsequently results in modulation of the membrane potential by regulation of ion channels, activation of the Na^+^/K^+^ exchanger and Ca^2+^ATPase, or regulation of actin cytoskeletal components anchored at the cell membrane. TH-activated MAPK, in turn, can rapidly translocate to the nucleus inducing serine phosphorylation of TRs, thereby resulting in the induction of angiogenesis or tumor cell proliferation. Nuclear targets for phosphorylated TRs include the transcription factors p53, STAT1a and STAT3

GFs are responsible for crosstalk between cell proliferation, migration, and adhesion. GFs bind to their specific receptor, cause cell-cell dissociation coupled to E-cadherin endocytosis and recycling back to the cell surface by several mechanisms [22, 23, 148] (Fig. 3). Co-regulation of cadherins and GF signaling is prominent in epithelial to mesenchymal transitions (EMT) and tumorigenesis [20, 88]. E-cadherins, in turn, modulate signal transduction by interacting with receptor tyrosine kinases, including the epidermal growth factor receptor (EGFR) [29, 163]. Among them, thyroid hormones (THs) modulate energy metabolism, having a great influence on growth and development by independent mechanisms [54]. An increasing number of studies show the THs action in different parallel signaling pathways via membrane receptors, cytoplasmic partners and thyroid hormone receptors (TRs) [72, 106]. TRs heterodimerize with retinoid X receptors (RXRs) and bind to T3 response elements (TRE) located within the genomic regions of target genes [212]. In the absence of T3, TRs interact with co-repressor proteins to inhibit TH-regulated target gene transcription (Fig. 4). Following T3 binding, co-repressors are displaced and co-activator proteins are recruited to the ligand-bound TR complex, so as to facilitate T3-dependent activation of the target genes. Complexity increases because the diploid organisms have THRA and THRB genes that encode the TRα and TRβ isoforms respectively, which are ubiquitously expressed [109, 175, 212, 213]. Moreover, depending on species, tissue or experimental systems, there are predominant TR cell isoforms, and each gene can generate different proteins using different promoters and/or alternative splicing [109, 175, 213]. In addition to functions mediated by TRs, THs also exert rapid non-genomic actions that are initiated at the cell membrane. For example, the αvβ3-integrin binds to THs, activating the mitogen-activated protein kinase (MAPK) cascade [45, 119] for modulating the membrane ion channels, Na^+^/K^+^ exchanger and Ca^2+^ATPase, as well as the actin cytoskeletal components [99] (Fig. 4).

Among genomic mechanisms, it is known that transcriptional repression of E-cadherins is mediated either by promoter CpG hypermethylation [60] or activation of repressors binded at E-boxes or brain-specific silencers, such as Snail and Slug [194], Twist [208] and E12/E47 (E2A gene product) [154]. In contrast, the miR-200 family directly targets repressors ZEB1 and ZEB2, promoting E-cadherin expression upregulation [103, 104] (Fig. 1). In hormonal signaling routes, it is known that the activated androgen receptor binds to promoter and also represses E-cadherin gene expression, promoting metastasis by EMT [120].

Conversely, our results suggest that T3-TRs promote E-cadherin, β- and α-catenin gene expression in vivo and EMT inhibition [56, 85]. E-cadherin locus up-regulation can be mediated by an enhancer element at intron 1 [12, 25, 69, 70]. In addition, Stemler Stemmler et al. [185] reveal a complex mechanism of gene regulation at mouse E-cadherin locus intron 2. While in differentiated epithelia intron 2 sequences are required both to initiate transcriptional activation and additionally to maintain E-cadherin expression during embryogenesis, the level of intron 2-dependent E-cadherin expression is relative to the tissues and developmental timing. Thus, early embryogenesis requires intron 2 for the onset of expression, but at later stages, a second mechanism initiates E-cadherin expression independently of intron 2, although for high-level expression the support of the intron 2 enhancer elements is still required. The onset of the second wave of expression was detected in the surface ectoderm differentiate to form skin, and in the gut endoderm (around E12.5). A locus control region (LCR), in turn, might influence gene activity for proper activation and downregulation, sited upstream of cadherin clusters [184, 185] with a vital role for the large intron 2 (Fig. 1). Striking, cadherin superfamily genes display a higher average total intron number and significantly longer introns than other genes and across the entire vertebrate lineage [144]. Particularly, the human genome has an uncommon high frequency of MIR and MaLR regulatory-associated repetitive elements at 5’-located introns, concomitant with increased *de novo* intronic transcription. Therefore, these intronic-specific sites may constitute targets of cadherin superfamily expression regulation, both in homeostasis and illness.

**Fig. 5:**
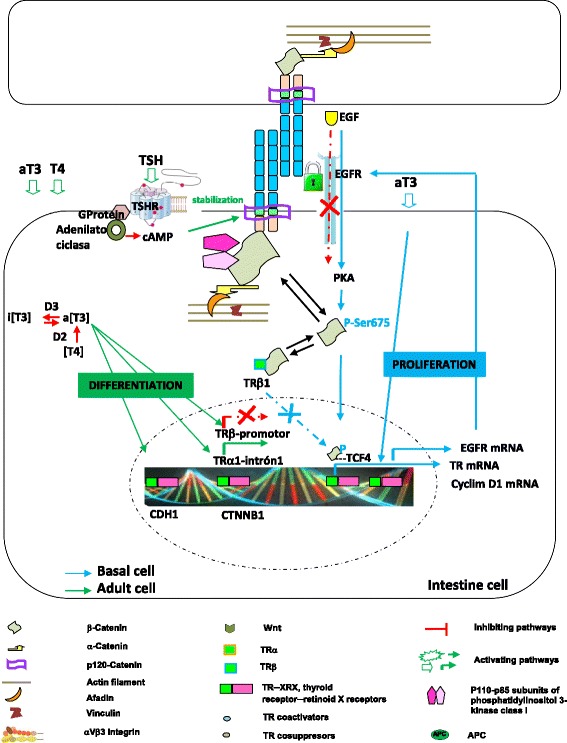
T3-control model of E-cadherin─β-catenin complex on gastrointestinal epithelial cells. THs and RTs function in cell proliferation, differentiation and apoptosis is not homogenous, because it depends strongly on physio-pathological context; that is, the cell-type, ontogeny (progenitor or differentiated cell) and health (normal or tumoral cell). However, it is possible to postulate that, while T3 induces epithelial basal cell proliferation via EGF-EGFR and cAMP-PKA signaling, T3 activates transcription of E-cadherin, β- and α-catenin genes in epithelial cells programmed to differentiate on pre-adult gut epithelia cells and inhibiting their EGF-EGFR dependent proliferative signal, as well as inhibiting their TH-integrin αvβ3 dependent migratory signals. In addition, because E-cadherin increases β-catenin sequestration at the plasma membrane, it then promotes cell differentiation by diminishing the β-catenin/TCF complex pool. At the same time, TSH-TSHR (receptor) signaling via cAMP stabilizes the assembly and retention of E-cadherin at the cell surface. TRα1 binds to β-catenin gene-intron 1-TRE (TRE-int1) in the intestine, increasing its expression via TH-binding. In parallel, TRα1 positively regulates the proliferation-controlling genes such as type D cyclins and c-myc, which are known targets of the Wnt/β-catenin. Increase of β-catenin/Tcf4, in turn, reduces the TRα1 transcriptional activity on its target genes. On the other hand, CTNNB1 transrepression is mediated by binding of the TRβ-RXR complexes to promoter TREs

Thus, searching for some of those physiological needs to sustain epithelial life, we have analyzed cell adhesion molecule response to 3,5,3’-triiodothyronine (T3), detecting morphometric evidences of gene upregulation exerted by T3 on E-cadherin, β- and α-catenin expression in different epithelial cell types of the metamorphosing anuran foregut [85]. Coincidentally, mouse β-catenin gene upregulation and transrepression by TH-TR [61, 158], as well as the impact of TH signaling in development, homeostasis and cancer susceptibility of mouse intestine [178] have been reported. T3-TRα1 binds directly to β-catenin gene-intron 1 specific TRE in the intestine, increasing its expression in an epithelial cell-autonomous way [158] (Fig. 5). This is parallel to positive regulation of proliferation-controlling genes, such as type D cyclins and c-myc, which are known targets of the Wnt/β-catenin pathway [158], synergizing Wnt pathway and inducing crypt cell proliferation and promoting tumorigenesis [179]. In contrast, CTNNB1 transrepression is mediated by binding of the TRβ-RXR complexes on TREs located in the human promoter between −807 and −772 (Fig. 5) [61]. Therefore, liganded TRβ acts as a tumor suppressor via inhibition of the expression of a potent tumor promoter, the CTNNB1 gene.

Even though TH signaling controls the proliferation of the intestinal epithelial progenitors in both amphibians and mammals, it has been suggested that TH control on the Wnt/β-catenin pathway does not appear to play a central role in amphibians [24, 178]. However, our in vivo experiments contradict this hypothesis, since we have detected early upregulation (24 hs) of E-cadherin, β- and α-catenin genes in the *Xenopus laevis* gut [56]. During metamorphic climax, larval cell apoptosis coexists with pre-adult (juvenile) cell proliferation and differentiation, and with cell-cell junction assembly─disassembly, that require a complex signal network to control tissular homeostasis. We found that T3 modulates epithelial adhesive potential during gut remodeling in *X. laevis* development, early and directly activating E-cadherin, β-catenin and α-catenin genes, and downstream, modulating small GTPases and other proteins involved in adhesive epithelial properties. Using INSECT2.0 web server to predict the occurrence of *Cis*-Regulatory Modules (CRMs) [150], we found putative TERs in *X. laevis* E-cadherin, β-catenin and α-catenin genes, but not in the p120-ctn gene [56]. Among evaluated small GTPases, gastrointestinal Rac1-mRNA levels significantly increased at 24 hs T3-treatment correlated with heightened “lamellipodia” or membrane protrusions. In contrast, at 5 days post T3-treatment RhoA-mRNA levels decrease while the important Rap1-mRNA increasing suggests that membrane Rap1-dependent E-cadherin recycling occurs at metamorphic climax end. Cdc42 and Arp2 actin nucleation protein became constant both during T3-induced and natural metamorphosis [56].

THs exert profound effects on tissues. Among them, cell-type dependent proliferation and differentiation both in mammalian skin [101, 113, 164, 195] and gut [158, 178], as in anuran kidney [84], skin [86] and gut [55, 56, 85]. These effects are regulated by phosphorilation levels of several partner proteins [86] and modulation of gen expression [56, 85]. Among the non-genomic effects of thyroid hormones, it appears that T3 activates PKA to in turn induce β-catenin nuclear translocation by phosphorylation at Ser675 site, thereby β-catenin modulates cyclin-D1 gene transcription and induces cell proliferation (Fig. 5) [53]. In contrast, TH causes astrocyte differentiation through both initial PKA activity and later by phospho-MAP kinase (p-MAPK or p-ERK) [58]. Postbirth, Schwann cell E-cadherin expression is highly regulated through cAMP-PKA activation for maintaining structural integrity [39]. Thus, E-cadherin can negatively regulate, in an adhesion dependent manner, the ligand-dependent activation of divergent classes of RTKs, by inhibiting their ligand-dependent activation in association with a decrease in receptor mobility and in ligand-binding affinity [163] (Fig. 5).

Therefore, the function of THs and their TRs in cell proliferation, differentiation and apoptosis is not homogenous, because it depends strongly on the physio-pathological context; that is, the cell-type, ontogeny (progenitor or differentiated cell) and health (normal or tumoral cell). Although TH-dependent processes are highly coordinated, in turn, the local requirements cannot be governed by global mechanisms, such as an alteration of the thyroid gland function and variations in plasma TH concentrations. Instead, they require tissue-specific regulation mediated by deiodinases. Thus, in mammals, T3 induces type 2 deiodinase (D2) and E-cadherin expression, which sequesters β-catenin and reduces both β-catenin/TCF complex and type 3 deiodinase (D3) activation levels. Consequently, the local active T3-level increases and promotes cell differentiation and reduces its oncogenic effects in intestinal cells (Fig. 5) [48]. Recently, this hypothesis has been again supported by Catalano and coworkers [28], who have found that increased intracellular TH concentration through D3 depletion induces cell differentiation and sharply mitigates tumor formation.

In this context, it is possible to postulate that during organ remodeling T3 induces epithelial stem cell proliferation via EGF-EGFR and cAMP-PKA signaling, and in parallel, T3 leads to repression of this via in a subgroup of these stem cells, and simultaneously increases E-cadherin, β- and α-catenin transcription via TH-RT to differentiate them to pre-adult (juvenile) gut epithelia cells (Fig. 5). In addition, the erichment of junctional E-cadherins sequestrates β-catenins, reducing their β-catenin nuclear translocalization and D3 disponibility, thereby strengthening cell differentiation.

## Conclusions

Unquestionably, E-cadherin is deeply involved in establishing cell polarity and differentiation, and thereby in the establishment and maintenance of tissue homeostasis during the development and the entire life of pluricellular organisms, mainly metazoans. Therefore, this transmembrane receptor is a potent suppressor of tumoral invasion and metastasis. Thus, E-cadherin must continuously react both at extra- and intracellular signals, some of which are classical, and very well known.

This review summarizes findings supporting the central role of thyroid hormones in controlling the availability and functionality of E-cadherin, through membrane, cytoplasmic and gene expression activities that regulate cell proliferation, differentiation, migration, and thereby epithelial homeostasis.

## Abbreviations

AJ: Adherens junctions
AQP3: Aquaporin 3
CD: Cytoplasmic domain
D2: Type 2 deiodinase
D3: Type 3 deiodinase
EC: Extracellular domain
EGFR: Epidermal growth factor receptor
EMT: Epithelial to mesenchymal transitions
GF: Growth factor
HAV: Histidine-alanine-valine
MAPK: Mitogen-activated protein kinase
O-GlcNAc: O-glycosylation
p120-ctn: p120-catenin
PALS: Protein Associated with Lin Seven 1
PI3K: Phosphatidylinositol-3-kinase
PIP3: Phosphatidylinositol-(3,4,5)-triphosphate
PS1: Presenilin 1
RT: Thyroid hormone receptor
RTK: Membrane-receptor tyrosine kinase
RXR: Retinoid X receptor
T3: 3,5,3’-triiodothyronine
TH: Thyroid hormone
TM: Trans-membrane domain
TRE: T3 response element
